# Association study of taste preference: Analysis in the Lithuanian population

**DOI:** 10.1002/fsn3.2401

**Published:** 2021-06-27

**Authors:** Ingrida Kavaliauskienė, Ingrida Domarkienė, Laima Ambrozaitytė, Lina Barauskienė, Raimonda Meškienė, Justas Arasimavičius, Algimantas Irnius, Vaidutis Kučinskas

**Affiliations:** ^1^ Department of Human and Medical Genetics Institute of Biomedical Science Faculty of Medicine Vilnius University Vilnius Lithuania

**Keywords:** association study, bitterness, lithuanian population, saltiness, SNP, sourness, sweetness, taste preference, umami

## Abstract

Taste has strong evolutionary basis in the sense of survival by influencing our behavior to obtain food/medicine or avoid poisoning. It is a complex trait and varies among individuals and distinct populations. We aimed to investigate the association between known genetic factors (673 SNPs) and taste preference in the Lithuanian population, as well as to determine a reasonable method for qualitative evaluation of a specific taste phenotype for further genetic analysis. Study group included individuals representing six ethnolinguistic regions of Lithuania. Case and control groups for each taste were determined according to the answers selected to the taste‐specific and frequency of specific food consumption questions. Sample sizes (case/control) for each taste are as follows: sweetness (55/179), bitterness (82/208), sourness (32/259), saltiness (42/249), and umami (96/190). Genotypes were extracted from the Illumina HumanOmniExpress‐12v1.1 arrays’ genotyping data. Analysis was performed using PLINK v1.9. We found associations between the main known genetic factors and four taste preferences in the Lithuanian population: sweetness—genes *TAS1R3, TAS1R2,* and *GNAT3* (three SNPs); bitterness—genes *CA6* and *TAS2R38* (six SNPs); sourness—genes *PKD2L1*, *ACCN2*, *PKD1L3,* and *ACCN1* (48 SNPs); and saltiness—genes *SCNN1B* and *TRPV1* (five SNPs). We found our questionnaire as a beneficial aid for qualitative evaluation of taste preference. This was the first initiative to analyze genetic factors related to taste preference in the Lithuanian population. Besides, this study reproduces, supports, and complements results of previous limited taste genetic studies or ones that lack comprehensive results concerning distinct (ethnic) human populations.

## Graphical abstract

The study explores Lithuanian ethnic group‐specific associations between taste preference and known genetic factors. Study reproduces and complements other studies' results in the taste genetics field. Additionally, a candidate questionnaire as a possible tool for taste preference identification is presented.

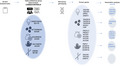

## 1 INTRODUCTION

Taste perception is part of flavor perception, which results primarily from the combination of three discrete senses: taste, somatosensation (touch, pain, and temperature), and olfaction. (Simon et al., 2006) Taste perception is strongly evolutionary in terms of physiological behavior such as obtaining a balance of electrolytes (saltiness), acquiring energy (sweetness), synthesizing proteins (umami), and avoiding poisonous (bitterness) or rotten (sourness) substances. (Purves et al., 2001).

Taste preference has a genetic background, and evidence suggests that the perception of different tastes is a polygenic or complex trait, though some taste phenotypes (traits) were thought to be inherited as Mendelian traits. (Guo & Reed, 2001) Taste‐related traits show different levels of heritability. Sweetness‐related traits such as the pleasantness, frequency of consumption, and craving for sweet foods show significant heritability (40%, 50%, and 31%, respectively). (Keskitalo et al., 2007) Heritability modeling on bitter stimuli showed a common genetic factor for quinine, caffeine, and sucrose octaacetate (22%–28%) and separate and specific genetic factors for propylthiouracil (72%) and quinine (15%). (Hansen et al., 2006) Heritability may include different types of genetic variation, unidentified genetic factors, environmental factors, and interaction between those factors, which are yet to be discovered. The first demonstration of how genetic variants shape interindividual differences in human taste sense was for the bitter taste receptor TAS2R38. (Kim et al., 2003) Individuals are referred to as tasters if being PAV (Pro at 49, Ala at 262, and Val at 296) haplotype and nontaster if being AVI (Ala at position 49, Val at 262, and Ile at 296) haplotype of the receptor. (Newcomb et al., 2012) Thus, the different haplotypes within the gene contribute to the intermediate phenotypes and thereby explain the nature of the quantitative trait. (Kim et al., 2003; Mennella et al., 2011) Genetic mapping and candidate gene association studies show that taste phenotypes are influenced by allelic variation of genes involved in both peripheral and central taste processing. (Bachmanov et al., 2013) Many genome‐wide association studies (GWASs) have been performed in the field of taste (Diószegi et al., 2019), but some of them lack the support of replication studies and the majority of them were performed in heterogeneous populations. This has led us to the knowledge of what is common for human populations from the genetic point of view. Nevertheless, when performing those studies, do we not miss what is specific and unique for different populations? There are a growing number of publications emphasizing the need to analyze various ethnic populations to better understand the architecture of genetic traits.

Behaviors mediated by the taste preference, particularly food choice and intake, are among the most important ones in terms of the health problems found in developed and developing societies. Taste, chemesthetic sensation, and responsible receptors’ genetics are relevant to study not only for the role in food preference, choice, and intake but also for the importance in human physiology and evolution. Function of these genes directly contributes overall health, indirectly health policy, food industry, and even global climate inevitably linked to the food industry. (Nolden & Feeney, 2020) Moreover, taste receptors and molecules are not exclusive to the oral cavity, but are found throughout the body and have multiple functions in tissues and organs, and have a role in a wide range of diseases. (ZhuGe et al., 2020) Having in mind genetic factors that contribute to the biology of taste preference, we should not forget the environmental component, which is significant in taste preference too. (Mennella & Beauchamp, 2005; Mennella et al., 2005) That is, a full understanding of taste is important for prevention of diseases such as hypertension, obesity, heart disease, diabetes, and some cancers. All of these diseases are strongly influenced by food choices that in turn are determined, in part, by how much we like (or dislike) the flavor of the food. (Beauchamp & Mennella, 2011) Genetic variation in taste preference influences human nutrition and health and could be used as a biomarker of predisposition to some diseases. (Bachmanov et al., 2013) As mentioned above, food choice arising from food preferences could be consciously controlled, in contrast to genetic factors. Thus, the accurate evaluation of food preference is important. It could be achieved by reasonable questionnaires. In these days of integrative and personalized medicine, it would be of great value for a clinician to have a simple tool that integrates as much as possible genotype and phenotype data.

The importance of taste research in ethnic populations and the demand to translate the results into clinical practice lead us to the aim of defining the genetic factors associated with the different taste preference in the Lithuanian population and of further evaluating the potential of using the questionnaire as a qualitative tool for food preference evaluation.

## 2 MATERIALS AND METHODS

### 2.1 Study group

Study group included unrelated individuals representing six ethno‐linguistic regions of Lithuania (West, North, and South Žemaitija and West, East, and South Aukštaitija). All self‐reported healthy individuals indicated at least three generations of Lithuanian ethnicity and residency in the same ethno‐linguistic region. Study participants were asked to fill in the dedicated questionnaire (see in a section “Questionnaire” below) and donate blood for DNA extraction and genotyping procedure. This is case–control genetic association study, so cases and controls were assigned as follows. Case and control groups for sweet, bitter, sour, umami, and salty food preference were determined according to the selected answers to the taste‐specific questions (for more information, see the *Questionnaire* section below). Sample sizes (case/control) for each taste modality and preference are as follows: sweetness (55/179), bitterness (82/208), sourness (32/259), saltiness (42/249), and umami (96/190). Only few individuals overlapped between case groups; for example, the same individual assigned to the sweet taste preference case group was also assigned to the salty taste preference case group. Besides, individuals qualified as cases for particular taste preference were assigned as controls for the other; for example, the case for a sweet taste preference was also a control for a bitter, sour, umami, and/ or sweet taste preferences.

### 2.2 Questionnaire

Twenty‐nine questions regarding certain tastes (sweetness, bitterness, sourness, saltiness, and umami) were asked in order to evaluate the food preference of every participant in the study. Food products were assigned to a certain taste group according to the literature that was reviewed. (Feeney et al., 2011; Garcia‐Bailo et al., 2009) There were six questions for sweetness, seven for bitterness, five for sourness, four for saltiness, and seven for umami (for the list of questions and possible choices, see File S1). There were three types of multiple‐choice questions: Type 1: the groups of food products representing a definite taste (sweetness, bitterness, sourness, saltiness, and umami); Type 2: periodicity of consumption of a product representing one of the five tastes; and Type 3: Yes/No questions reflecting the consumption of extra products (such as sugar, salt, or pepper) to enhance a certain taste. Individuals were grouped according to their preference for sweet, sour, salty, bitter, or umami flavors. If the sweet product group was chosen, that is, carrots, potatoes, or beetroot, while answering Type 1 questions, the individual was put into the sweet taste case group. If while answering Type 2 questions an individual admitted using certain products, that is, sweet ones, 3–5 times per week or more, that person was added to the sweet taste case group. If while answering Type 3 questions a subject indicated that he used certain taste‐enhancing products, that is, sugar, that person would be placed into the sweet taste case group. The other four taste case groups were determined in the same manner. Individuals were listed as controls if answering Type 1 questions with other food preference than the tested one, Type 2 questions with the less frequent consumption of the food tested for food preference, and (or) Type 3 questions with a contrary answer.

### 2.3 Genome‐wide genotyping

Genomic DNA was extracted from venous blood using either the phenol–chloroform extraction method or automated nucleic acid purification using paramagnetic particles (Freedom EVO^®^ Nucleic Acid Purification Workstation). The quality and quantity of purified genomic DNA were evaluated with a spectrophotometer (NanoDrop^®^ ND‐1000 Spectrophotometer). Genome‐wide genotyping following manufacturer's protocols was performed using high‐density Illumina HumanOmniExpress‐12v1.1 arrays (719,666 SNPs) on an Illumina HiScan^™^SQ system.

### 2.4 Data analysis

Primary data quality control analysis was performed using GenomeStudio v2011.1 (Illumina^®^ GenomeStudio 2011, Illumina, Inc. 2003–2011). PLINK v1.9 (Purcell et al., 2007) software was used for the secondary data analysis: filtering of SNPs and individuals, calculation of minor allele frequency (MAF), Hardy–Weinberg equilibrium (HWE), case–control association under different genetic models, and permutation test.

Association with five different phenotypes (taste preference for sweetness, bitterness, sourness, saltiness, and umami) was tested using the chi‐square test of independence or Fisher's exact test depending on the minimal number of genotypes (the minimal number of genotypes was five for the chi‐square test of independence and 0 for the Fisher's exact test). Five different genetic models (basic: genotypic, allelic and additive: Cochran–Armitage trend, dominant, and recessive) were used to evaluate association between phenotypes and known genetic factors. Genetic variants or SNPs (hereafter variants) of known candidate taste preference genes analyzed in this study are provided in Table 1. Covariates such as age, sex, body mass index (BMI), family history, or other environmental factors were not included in the analysis. A significance level (α) of 0.05 was set for this study. Permutation procedure (*n* = 10,000) was used to obtain empirical *p*‐value for the chi‐square test of independence or Fisher's exact test.

**TABLE 1:** Variants of candidate genes of taste

Genes	Reference	Number of SNPs analysed	Number of subjects (cases/controls)
Sweetness			
*TAS1R2*	Bachmanov et al., 2011)	7	55/179
*TAS1R3*	Bachmanov et al., 2011)	1	
*DRD2*	Eny et al., 2009)	19	
*GNAT3*	Fushan et al., 2010)	15	
Total:		42	
Bitterness			
*TAS2R38*	Kim et al., 2003)	2	82/208
*CA6*	Padiglia et al., 2010)	16	
*TAS2R16*	Hayes et al., 2011)	2	
*TAS2R19*	Hayes et al., 2011)	1	
*RGS21*	Cohen et al., 2012)	9	
Total:		30	
Sourness			
*PKD1L3*	Ishimaru et al., 2006)	25	32/259
*PKD2L1*	Ishimaru et al., 2006)	15	
*ACCN1*	Huque et al., 2009)	453	
*ACCN2*	Huque et al., 2009)	10	
*ACCN3*	Huque et al., 2009)	3	
*ACCN4*	Huque et al., 2009)	8	
Total:		514	
Saltiness			
*TRPV1*	Dias et al., 2013)	23	42/249
*SCNN1A*	Dias et al., 2013)	8	
*SCNN1B*	Dias et al., 2013)	21	
*SCNN1G*	Dias et al., 2013)	11	
Total:		63	
Umami			
*TAS1R1*	Shigemura et al., 2009)	8	96/190
*TAS1R3*	Shigemura et al., 2009)	1	
*GNAT3*	Kinnamon, 2005)	15	
Total:		24	

## 3 RESULTS

Analyzed variants were frequent in different populations and with different functions: synonymous or nonsynonymous (missense) in different genomic regions (introns or exons). Rare variants and variants from several candidate genes were not analyzed, since the genome‐wide genotyping array did not include them.

HWE testing for all variants was conducted in three groups, that is, only cases, only controls, and combined case and control group. Because the genome‐wide genotyping call rate was 0.97 and higher for all samples, variants with HWE *p* < .001 in any group were removed from further analysis. As a result, 46 variants were excluded from the analysis (sweetness—0 variants; bitterness—8 variants; sourness—30 variants; umami—4 variants; and saltiness—4 variants).

Statistically significant (*p* < .05) associations between SNPs and different taste preferences were shown: 3 for sweetness; 6 for bitterness; 48 for sourness; 5 for saltiness; and 1 nearly statistically significant association for umami.

### 3.1 Variants associated with sweet taste preference

Three variants were significantly associated with sweet taste preference (Table 2, significant *p*‐values in bold): *TAS1R3* gene SNP rs35424002 (NM_152228.1:c.*142G>A); *TAS1R2* gene SNP rs9988418 (NM_152232.2:c.2513G>A, NP_689418.2:p.(Arg838Lys)); and *GNAT3* gene SNP rs10230573 (NM_001102386.1:c.118+56T>C).

**TABLE 2:** Statistically significant results of the analysis of the association between sweet taste preference and SNPs

Chr^a^	Gene	SNP	A1^b^	A2^c^	Test	Aff^d^	Unaff^e^	*p*	*p* ^f^	Fisher's *p*	Fisher's *p* ^g^	OR
1	*TAS1R3*	rs35424002	A	G	Geno^h^	1/7/47	0/13/166	–		.101		2.365
					Trend^i^	9/101	13/345	.054		.054		
					Allelic^j^	9/101	13/345	.**049**	.057	.068	.066	
					Dom^k^	8/47	13/166	–		.109		
					Rec^l^	1/54	0/179	–		.235		
1	*TAS1R2*	rs9988418	A	G	Geno	0/4/51	0/2/177	–		.029		6.717
					Trend	4/106	2/356	.**012**		.012		
					Allelic	4/106	2/356	.**012**	.016	.029	.017	
					Dom	4/51	2/177	–		.029		
					Rec	0/55	0/179	–		1.000		
7	*GNAT3*	rs10230573	A	G	Geno	5/22/28	31/80/68	.151		.170		0.624
					Trend	32/78	142/216	.052		.052		
					Allelic	32/78	142/216	.**045**	.096	.055	.089	
					Dom	27/28	111/68	.088		.117		
					Rec	5/50	31/148	.139		.199		

Note

a—chromosome; b—allele 1; c—allele 2; d—distribution of alleles or genotypes in the case group; e—distribution of alleles or genotypes in the control group; f—empirical *p*‐value for chi‐square test of independence (permutation test based on the most significant result of allelic dominant and recessive models); g—empirical *p*‐value for Fisher's exact test (permutation test based on the most significant result of allelic dominant and recessive models); h—basic model: genotypic; i—additive model: Cochran–Armitage trend; j—basic model: allelic; k—additive model: dominant; l—additive model: recessive.

### 3.2 Variants associated with bitter taste preference

Six variants were shown to be significantly associated with bitter taste preference (Table 3, significant *p*‐values in bold): *CA6* gene SNPs rs2274327 (NM_001215.3:c.164C>T, NP_001206.2:p.(Thr55Met)), rs2274328 (NM_001215.3:c.202A>C, NP_001206.2:p.(Met68Leu)), rs1832262 (NM_001215.3:c.502‐1741T>C), and rs3765964 (NM_001215.3:c.845‐260G>A) and *TAS2R38* gene SNPs rs10246939 (NM_176817.4:c.886A>G, NP_789787.4:p.(Ile296Val)) and rs1726866 (NM_176817.4:c.785T>C, NP_789787.4:p.(Val262Ala)). Four more variants showed an association with bitter taste under basic allelic or genotypic models but were eliminated due to discrepancy from HWE (*p* < .001).

**TABLE 3:** Statistically significant results of the analysis of the association between bitter taste preference and SNPs

Chr^a^	Gene	SNP	A1^b^	A2^c^	Test	Aff^d^	Unaff^e^	*p*	*p* ^f^	Fisher's *p*	Fisher's *p* ^g^
1	*CA6*	rs2274327	A	G	Geno^h^	9/27/46	19/112/77	.**005**		.**004**	
					Trend^i^	45/119	150/266	.**040**		.**040**	
					Allelic^j^	45/119	150/266	.**048**	.007	.051	.007
					Dom^k^	36/46	131/77	.**003**		.**004**	
					Rec^l^	9/73	19/189	.633		.661	
		rs2274328	C	A	Geno	15/32/34	34/113/60	.055		.057	
					Trend	62/100	181/233	.226		.226	
					Allelic	62/100	181/233	.234	.071	.260	.070
					Dom	47/34	147/60	.**035**		.**037**	
					Rec	15/66	34/173	.671		.728	
		rs1832262	A	G	Geno	23/31/28	45/115/48	.**025**		.**024**	
					Trend	77/87	205/211	.612		.612	
					Allelic	77/87	205/211	.614	.098	.645	.134
					Dom	54/28	160/48	.054		.075	
					Rec	23/59	45/163	.246		.282	
		rs3765964	A	G	Geno	17/34/31	19/115/74	.**013**		.**016**	
					Trend	68/96	153/263	.273		.273	
					Allelic	68/96	153/263	.296	.014	.298	.015
					Dom	51/31	134/74	.722		.786	
					Rec	17/65	19/189	.**007**		.**010**	
7	*TAS2R38*	rs10246939	G	A	Geno	13/44/25	27/90/91	.116		.115	
					Trend	70/94	144/272	.071		.071	
					Allelic	70/94	144/272	.070	.076	.085	.090
					Dom	57/25	117/91	.**038**		.**046**	
					Rec	13/69	27/181	.523		.571	
		rs1726866	G	A	Geno	13/44/25	27/90/91	.116		.115	
					Trend	70/94	144/272	.071		.071	
					Allelic	70/94	144/272	.070	.076	.085	.090
					Dom	57/25	117/91	.**038**		.**046**	
					Rec	13/69	27/181	.523		.571	

Note

a—chromosome; b—allele 1; c—allele 2; d—distribution of alleles or genotypes in the case group; e—distribution of alleles or genotypes in the control group; f—empirical *p*‐value for chi‐square test of independence (permutation test based on the most significant result of allelic dominant and recessive models); g—empirical *p*‐value for Fisher's exact test (permutation test based on the most significant result of allelic dominant and recessive models); h—basic model: genotypic; i—additive model: Cochran–Armitage trend; j—basic model: allelic; k—additive model: dominant; l—additive model: recessive.

### 3.3 Variants associated with sour taste preference

Analysis showed 41 variants of the *ACCN1* gene statistically significantly associated with sour taste preference under basic genotypic and/or allelic models and additive recessive and/or dominant models (Table S2). Seven significantly associated variants in other genes are provided in Table 4 (significant *p*‐values in bold): *PKD2L1* gene SNP rs12360462 (NM_016112.2:c.350‐4085T>C); *ACCN2* gene SNPs rs835592 (NM_001095.3:c.558+7094T>C), rs2272391 (NM_001095.3:c.710‐153A>G), and rs7305558 (NM_001095.3:c.1052‐308G>A); and *PKD1L3* gene SNPs rs16973500 (NM_181536.1:c.4927‐1110G>A), rs9925415 (NM_181536.1:c.1777G>A, NP_853514.1:p.(Val593Met)), rs9928317 (NM_181536.1:c.586‐1755A>C), and rs4788592 (NM_181536.1:c.585+1238G>A).

**TABLE 4:** Statistically significant results of the analysis of the association between sour taste preference and SNPs (*ACCN1* variants excluded)

Chr^a^	Gene	SNP	A1^b^	A2^c^	Test	Aff^d^	Unaff^e^	*p*	*p* ^f^	Fisher's *p*	Fisher's *p* ^g^
10	*PKD2L1*	rs12360462	G	A	Geno^h^	11/12/9	49/122/87	.128		.142	
					Trend^i^	34/30	220/296	.122		.122	
					Allelic^j^	34/30	220/296	.111	.095	.141	.102
					Dom^k^	23/9	171/87	.526		.691	
					Rec^l^	11/21	49/209	.**043**		.061	
12	*ACCN2*	rs835592	G	A	Geno	8/16/8	31/137/90	.109		.118	
					Trend	32/32	199/317	.063		.063	
					Allelic	32/32	199/317	.078	.090	.080	.092
					Dom	24/8	168/90	.265		.324	
					Rec	8/24	31/227	.**042**		.054	
12		rs2272391	A	G	Geno	9/15/8	34/146/78	.081		.103	
					Trend	33/31	214/302	.098		.098	
					Allelic	33/31	214/302	.124	.065	.141	.055
					Dom	24/8	180/78	.541		.682	
					Rec	9/23	34/224	.**025**		.034	
12		rs7305558	A	G	Geno	1/1/0	0/12/23	–		.**020**	
					Trend	3/1	12/58	.**003**		.**003**	
					Allelic	3/1	12/58	.**005**	.005	.**030**	.014
					Dom	2/0	12/23	–^m^		.137	
					Rec	1/1	0/35	–		.054	
16	*PKD1L3*	rs16973500	A	G	Geno	1/16/15	16/74/168	–		.**050**	
					Trend	18/46	106/410	.179		.179	
					Allelic	18/46	106/410	.163	.188	.195	.083
					Dom	17/15	90/168	–		.053	
					Rec	1/31	16/242	–		.704	
16		rs9925415	A	G	Geno	2/22/8	69/116/73	–		.**010**	
					Trend	26/38	254/262	.204		.204	
					Allelic	26/38	254/262	.194	.229	.233	.017
					Dom	24/8	185/73	–		.835	
					Rec	2/30	69/189	–		.**009**	
16		rs9928317	C	A	Geno	2/20/8	69/110/71	–		.**016**	
					Trend	24/36	248/252	.174		.174	
					Allelic	24/36	248/252	.160	.165	.173	.023
					Dom	22/8	179/71	–		1.000	
					Rec	2/28	69/181	–		.**013**	
16		rs4788592	A	G	Geno	1/20/11	51/119/88	–		.**031**	
					Trend	22/42	221/295	.200		.200	
					Allelic	22/42	221/295	.196	.215	.227	.044
					Dom	11/21	170/88	–		1.000	
					Rec	3/29	51/207	–		.**025**	

Note

a—chromosome; b—allele 1; c—allele 2; d—distribution of alleles or genotypes in the case group; e—distribution of alleles or genotypes in the control group; f—empirical *p*‐value for chi‐square test of independence (permutation test based on the most significant result of allelic dominant and recessive models); g—empirical *p*‐value for Fisher's exact test (permutation test based on the most significant result of allelic dominant and recessive models); h—basic model: genotypic; i—additive model: Cochran–Armitage trend; j—basic model: allelic; k—additive model: dominant; l—additive model: recessive; m—no data available.

### 3.4 Variants associated with salty taste preference

Our analysis identified five variants statistically significantly associated with salty taste preference (Table 5, significant *p*‐values in bold): *SCNN1B* gene SNPs rs12162045 (NM_000336.2:c.‐9+17985G>A) and rs152733 (NM_000336.2:c.312‐1444T>C); and *TRPV1* gene SNPs rs877610 (NM_018727.5:c.2157G>A, NP_061197.4:p.(Lys719=)), rs8078936 (NM_018727.5:c.1780+24G>A), and rs150908 (NM_018727.5:c.1477‐547C>T).

**TABLE 5:** Statistically significant results of the analysis of the association between salty taste preference and genetic variants

Chr^a^	Gene	SNP	A1^b^	A2^c^	Test	Aff^d^	Unaff^e^	*p*	*p* ^f^	Fisher's *p*	Fisher's *p* ^g^
16	*SCNN1B*	rs12162045	A	G	Geno^h^	0/10/32	12/90/147	–^m^		.075	
					Trend^i^	10/74	114/384	.**021**		.**021**	
					Allelic^j^	10/74	114/384	.**023**	.025	.**021**	.034
					Dom^k^	10/32	102/147	–		.**040**	
					Rec^l^	0/42	12/237	–		.226	
16		rs152733	G	A	Geno	37,953	1/58/188	–		.**013**	
					Trend	17/67	60/434	.**040**		.**040**	
					Allelic	17/67	60/434	.**044**	.046	.**055**	.016
					Dom	14/28	59/188	–		.248	
					Rec	3/39	1/246	–		.**010**	
17	*TRPV1*	rs877610	A	G	Geno	0/0/42	3/23/223	–		.093	
					Trend	0/84	29/469	.**036**		.**036**	
					Allelic	0/84	29/469	.**023**	.029	.**014**	.018
					Dom	0/42	26/223	–		.**020**	
					Rec	0/42	3/246	–		1.000	
17		rs8078936	A	G	Geno	11/14/17	26/125/98	.**010**		.**015**	
					Trend	36/48	177/321	.191		.191	
					Allelic	36/48	177/321	.198	.014	.221	.020
					Dom	25/17	151/98	.891		1.000	
					Rec	11/31	26/223	.**005**		.**010**	
17		rs150908	A	G	Geno	8/28/6	63/115/71	.**041**		.**048**	
					Trend	44/40	241/257	.502		.502	
					Allelic	44/40	241/257	.499	.101	.556	.099
					Dom	13,302	178/71	.053		.059	
					Rec	8/34	63/186	.383		.442	

Note

a—chromosome; b—allele 1; c—allele 2; d—distribution of alleles or genotypes in the case group; e—distribution of alleles or genotypes in the control group; f—empirical *p*‐value for chi‐square test of independence (permutation test based on the most significant result of allelic dominant and recessive models); g—empirical *p*‐value for Fisher's exact test (permutation test based on the most significant result of allelic dominant and recessive models); h—basic model: genotypic; i—additive model: Cochran–Armitage trend; j—basic model: allelic; k—additive model: dominant; l—additive model: recessive; m—no data available.

### 3.5 Variants associated with umami taste preference

Nearly statistically significant results (Table 6) were identified for *TAS1R1* gene SNP rs12565181 (NM_138697.3:c.191+4921G>A).

**TABLE 6:** Nearly statistically significant results of the analysis of the association between umami taste preference and genetic variant

Chr^a^	Gene	SNP	A1^b^	A2^c^	Test	Aff^d^	Unaff^e^	*p*	*p* ^f^	Fisher's *p*	Fisher's *p* ^g^
1	*TAS1R1*	rs12565181	A	G	Geno^h^	2/25/69	2/33/154	–^m^		.129	
					Trend^i^	29/163	37/341	.062		.062	
					Allelic^j^	29/163	37/341	.061	.063	.072	.081
					Dom^k^	27/69	35/154	–		.070	
					Rec^l^	2/94	2/187	–		.605	

Note

a—chromosome; b—allele 1; c—allele 2; d—distribution of alleles or genotypes in the case group; e—distribution of alleles or genotypes in the control group; f—empirical *p*‐value for chi‐square test of independence (permutation test based on the most significant result of allelic dominant and recessive models); g—empirical *p*‐value for Fisher's exact test (permutation test based on the most significant result of allelic dominant and recessive models); h—basic model: genotypic; i—additive model: Cochran–Armitage trend; j—basic model: allelic; k—additive model: dominant; l—additive model: recessive; m—no data available.

## 4 DISCUSSION

Sweetness is one of the most studied tastes. Statistically significant association of two variants, rs35424002 in 3'UTR of the *TAS1R3* gene and rs9988418 in the coding region of the *TAS1R2* gene, supports the results of previous studies in which the mammalian sweet taste heteromeric G‐protein‐coupled receptor complex (TAS1R3/TAS1R2) was proved to be the major player in the sense of sweetness, (Zhao et al., 2003) and variants found upstream of *TAS1R3* and *TAS1R2* genes’ sequences were associated with human taste sensitivity to sucrose (Fushan et al., 2010). The odds ratio for rs35424002 (OR = 2.365) was lower than the odds ratio for rs9988418 (OR = 6.717), but both variants showed significant impact on the taste phenotype. Difference in ORs could be explained by the nature of the molecular role played by a particular variant in either regulatory changes in TAS1R3 or conformational changes in the TAS1R2 protein. A TAS1R3 query in the STRING database (Jensen et al., 2009) alongside TAS1R1 and TAS1R3 revealed GNAT3 as another player in sweet taste pathway. This alpha subunit is further downstream of the sweet taste signal transduction cascade as it binds to the cell surface receptors through cGMP phosphodiesterase. (Kinnamon, 2005) The statistically significant association of rs10230573 in the *GNAT3* gene confirms the involvement of this pathway in the sweet taste signal. The small effect size of the rs10230573 (OR = 0.6241) could be because the alpha subunit in the sweet taste signal transduction pathway is not specific and has a role in different taste pathways too. (Jang et al., 2007; Li et al., 2002).

Bitterness is another well‐studied taste. This study confirmed the statistically significant associations of rs2274327, rs2274328, rs1832262, and rs3765964 in the *CA6* gene, two coding and two intronic, respectively, and coding rs10246939 and rs1726866 in the *TAS2R38* gene. The product of the *CA6* gene is known as the gustin, or carbonic anhydrase 6 (CA VI), isozyme of the carbonic anhydrase secreted in saliva and milk. (Pastorekova et al., 2004) CA VI was found to be associated with bitter taste, and *CA6* SNP rs2274327 has been linked with picky eating behavior in preschool‐age children (Cole et al., 2017) and implicated in taste bud function and salivary buffer capacity (Peres et al., 2010). It was postulated that CA VI may be a mechanistic link between 6‐n‐propylthiouracil tasting and fungiform taste papilla density and maintenance, (Melis et al.,) but a later study did not detect such an association (Feeney & Hayes, 2014). Thus, the role of this protein and interactions with other proteins is ambiguous and obscure. KEGG Database (Kanehisa & Goto, 2000) Pathway hsa00910 (Release 1/9/20) reveals enzyme CA VI as a participant in the nitrogen metabolism related to cyanate as an assistant reaction with bicarbonate and carbon dioxide. It is also known that cyanogenic glycosides present in plants have a bitter taste and if eaten without processing could be hydrolyzed to cyanide. According to the existing knowledge of cyanide metabolism, it might be transformed into cyanate (Petrova Simeonova & Fishbein, 2004) and here, hypothetically, comes the time for CA VI to act. The association of *TAS2R38* gene variants (OR = 1.407 for both variants) supports previous studies finding it to be a gene important in phenylthiocarbamide sense. (Kim et al., 2003) The *TAS2R38* gene encodes a receptor, the first element in the bitter taste pathway. Subsequent protein coupling this receptor is G‐protein gustducin dissociates its α, Gnat3, and βγ subunits and further downstream the canonical T2R signal transduction cascade. (Lu et al., 2017) It is known that TAS2R38 could be co‐expressed with GNAT3 in some tissues, making GNAT3‐dependent signal transduction possible. (Imai et al., 2020) Still, this study did not find the association between *GNAT3* variants (results not shown) and bitter taste preference. Variants of *RGS21* (regulator of G‐proteins), (Cohen et al., 2012) and *TAS2R16* and *TAS2R19* (TAS2 family receptors) genes were not associated with bitter taste preference either. These results imply the need for further research on the role of other G‐proteins and their regulators in bitter taste pathway.

Sour taste stimuli are thought to be mainly acids (H^+^), and the mechanism of signal transduction is different than it is with sweet, bitter, or umami tastes. Sour and salty taste stimuli (Na^+^ or K^+^) are transported into the taste cells through ion channels rather than receptors as sweet, bitter, and umami stimuli are. (Roper, 2007) Instead of transporting molecules, ion channels translate chemical signals into electrophysiological signals. The polycystic kidney disease 1 and polycystic kidney disease 2–like proteins PKD2L1 and PKD1L3 have been identified as sour taste‐related receptors in human taste cells (Ishimaru et al., 2006), and potential ion‐channel OTOP1 was present in taste cells in mouse that express *Pkd2l1*. (Tu et al., 2018) In our study, statistically significant association between sour taste preference and four variants (noncoding rs12360462 *PKD2L1* gene; rs9925415, rs9928317, and rs4788592 *PKD1L3* genes, only rs9928317 coding) was observed. This association of variants for both genes supports the involvement of these proteins in sour taste signal as heteromeric/homomeric complexes or separate parts. Statistically significant association of another 44 variants in *ACCN2* (rs835592, rs2272391, and rs7305558 with the highest OR = 14.5 for rs7305558) and *ACCN1* (full list in Table S2) genes was detected. This supports the assumption that acid taste pathway can start by several different channels in the taste cells. (Huque et al., 2009) It is likely that not independent variants but several haplotypes of the *ACCN1* gene are responsible for variation in sour taste preference.

It is known that a heterodimer of TAS1R1 and TAS1R3 (TAS1R1/TAS1R3) functions as an umami taste receptor in humans. (Li et al., 2002) Variants of the *TAS1R1* and *TAS1R3* genes were studied, but only one variant (rs2274327, *TAS1R1* gene, OR = 1.64) showed nearly statistically significant association with umami taste preference. Association was not found with the *TAS1R3* SNP possibly due to the small number of *TAS1R3* SNPs analyzed (only one). Moreover, there was no association found with *GNAT3*. This could be because GNAT3 is involved in more than one different taste signal transduction pathway and is less specific.

Animal models showed that the sodium‐specific and amiloride‐sensitive epithelial sodium channel (ENaC) and the transient receptor potential cation subfamily V member 1 (TRPV1) amiloride insensitive channel are candidates for the pathway of salty taste. (Bigiani, 2020) The results of this study support this evidence as association between variants (noncoding rs12162045 and rs152733 in *SCNN1B* gene, OR = 0.455 and OR = 1.835, respectively; synonymous rs877610, noncoding rs8078936 and rs150908 in *TRPV1* gene) and saltiness preference was observed. There is a lack of evidence for *SCNN1A* and *SCNN1G* genes being associated with saltiness and the involvement of these proteins in salty taste pathway in humans. Our study did not identify such an association either.

The results of this study reveal only a fragment of the full spectra network elements in complex signal transduction pathways for different tastes. The food preference too is a very complex trait and depends not only on biological factors (such as age, sex, genetics), but also on culture, socio‐economic status. (Davide et al., 2017; Mennella & Beauchamp, 2005).

It became clear that science must unravel what was left behind by the GWASs in admixture populations. The studies on specific ethnic groups and their genetic differences in taste perception already began. If we looked at the genetic structure of the Lithuanian population, it would fall within the range of European populations. (Nelis et al., 2009) Lithuanians were found to be homogenous and genetically close to neighboring populations. (Kasperaviciūte et al., 2004; Nelis et al., 2009) Nevertheless, it was confirmed that Lithuanian population preserved one of the highest proportions of western, Scandinavian, and eastern hunter–gather ancestry components found in European populations and also that of a steppe Early to Middle Bronze Age pastoralists, which show the genetic distinctiveness of the Lithuanian population. (Urnikyte et al., 2019) This is one of the reasons why the Lithuanian population is unique of studying and why some genetic associations found in other studies do not reproduce. Nevertheless, our study was able to reproduce some of the valuable results of other taste genetics studies. This is quite an important result in the context of the huge reproducibility problem of scientific results (Amaral et al., 2019; Open Science Collaboration, 2015). Besides, the results can indicate that the questionnaire used in the study proved its value and might be a useful tool for a clinician for food preference evaluation, but further validity assessment is needed.

## 5 CONCLUSION

The results of the study reproduced associations of the main known genetic factors and four of the five tastes: sweetness—the genes *TAS1R3, TAS1R2,* and *GNAT3* (three variants); bitterness—the genes *CA6* and *TAS2R38* (six variants); sourness—the genes *PKD2L1*, *ACCN2*, *PKD1L3,* and *ACCN1* (48 variants); and saltiness—the genes *SCNN1B* and *TRPV1* (five variants). Most of the associations show genetic factors that are the primary taste signal transduction pathway players in the taste bud cells (G‐protein‐binding receptors or ion channels), since they are very specific to particular tastes. Genetic factors encoding proteins that are further downstream of the pathway usually are not that specific and that could be one of the reasons why this particular study design failed to find the associations. Other reasons might include the difference in genetic structure of the population, the sample size of the study, nongenetic factors that contribute to the food preference, and structure and content of the questionnaire. The lack of specific questions provides no chance to detect any significant association, as occurred in this study while analyzing the umami taste case. This study also supports some results of a few studies and complements ones that lack comprehensive results on distinct (ethnic) human populations. Finally, we found our questionnaire (based on very specific questions about nutritional habits) a beneficial aid for *qualitative* evaluation of taste preference. To reliably classify individuals for food preference, there must be a sufficient number of questions including all food groups and specifying certain tastes.

## CONFLICT OF INTEREST

The authors have no conflict of interest to declare.

## AUTHOR CONTRIBUTIONS

**Ingrida Kavaliauskienė:** Conceptualization (supporting); Data curation (equal); Formal analysis (lead); Investigation (lead); Methodology (lead); Project administration (lead); Writing‐original draft (equal); Writing‐review & editing (equal). **Ingrida Domarkienė:** Data curation (equal); Formal analysis (supporting); Writing‐original draft (equal); Writing‐review & editing (lead). **Laima Ambrozaitytė:** Data curation (equal); Formal analysis (supporting); Project administration (supporting); Writing‐review & editing (equal). **Lina Barauskienė:** Methodology (supporting); Writing‐original draft (supporting); Writing‐review & editing (supporting). **Raimonda Meškienė:** Investigation (supporting); Writing‐review & editing (supporting). **Justas Arasimavičius:** Data curation (equal); Software (lead); Writing‐review & editing (supporting). **Algimantas Irnius:** Conceptualization (supporting); Methodology (supporting); Supervision (supporting); Writing‐review & editing (supporting). **Vaidutis Kučinskas:** Conceptualization (lead); Data curation (lead); Funding acquisition (lead); Project administration (supporting); Resources (lead); Supervision (lead); Writing‐review & editing (supporting).

## STUDIES INVOLVING HUMAN SUBJECTS

The study conforms to the Declaration of Helsinki, US, and/or European Medicines Agency Guidelines for human subjects. Study's protocols and procedures were ethically reviewed and approved by a recognized ethical body (the Vilnius Regional Biomedical Research Ethics Committee (Permission No. 158200‐05‐329‐79), Lithuania). Informed written consent was obtained and documented from each individual included in the study.

## Supporting information

Supplementary Material

Table

## Data Availability

All necessary data is provided in the article. The raw data that support the findings of this study is available from the corresponding author upon reasonable request.
