# Supplementary material for: Association study of taste preference: Analysis in the Lithuanian population
Source: Food Sci Nutr. 2021 Jun 27;9(8):4310–21. doi: 10.1002/fsn3.2401 (PMC8358374; doi:10.1002/fsn3.2401)
Supplement: Supplementary file 1 — Supplementary Material [file FSN3-9-4310-s002.docx]

**Groups of questions and possible answers.** Answers in *italics* rated individuals as preferring particular taste (of each taste group).

**SWEET TASTE**

**1. Which of these vegetable groups do you find the most tasty (mark one):**

1 broccoli, brussel sprouts, cabbages

*2 potatoes, carrots, beetroots*

3 spinach, sorrel, marinated vegetables

4 sour pickles, sour marinated tomatoes

**2. How often do you consume carrots:**

1 never

2 less than once a week

3 1-2 times per week

*4 3-5 times per week*

*5 every day*

*6 a few times a day*

**3. Which of this fruit do you prefer:**

1 bitter ones (grapefruit group)

*2***** *sweet ones (pears, bananas, sweet apples)*

3 sour ones (oranges, currants, cherries, lemons)

**4. Which of the listed product groups do you prefer:**

*1***** *milk and sweet diary products (sweet cheese, sweet cottage cheese, sweet yoghurt);*

2 sour dairy products (kefir, butter milk, natural yoghurt);

3 fermented cheese

1. **Do you drink coffee:**

*1 yes*

2 no

**If yes, is it: 5.1** *1 with sugar* 2 without sugar

**6. Do you drink green tea:**

*1 yes*

2 no

**If yes, is it:**  **6.1** *1 with sugar* 2 without sugar

**BITTER TASTE**

**1. Which of these vegetable groups do you find the most tasty (mark one):**

*1* *broccoli, brussel sprouts, cabbages*

2**** potatoes, carrots, beetroots

3 spinach, sorrel, marinated vegetables

4 sour pickles, sour marinated tomatoes

**2. How often do you consume cabbages:**

1 never

2 less than once a week

3 1-2 times per week

*4 3-5 times per week*

*5 every day*

*6 a few times a day*

**3. Which of this fruit do you prefer:**

*1 bitter ones (grapefruit group)*

2 sweet ones (pears, bananas, sweet apples)

3 sour ones (oranges, currants, cherries, lemons)

**4. Which salad sauce do you find the most tasty (mark one):**

1 soy sauce

2 lemon juice

3 salt

*4 pepper*

1. **Do you add extra pepper to dishes while eating:**

1 never

*2 a little, if the dish is not spicy enough*

*3 almost always, without even tasting*

1. **Do you drink coffee:**

*1 yes*

2 no

**If yes, is it: 6.1** 1 with sugar 2 *without sugar*

**7. Do you drink green tea:**

*1 yes*

2 no

**If yes, is it:**  **7.1** 1 with sugar 2 *without sugar*

**SALTY TASTE**

**1. Which of these vegetable groups do you find the most tasty (mark one):**

1 broccoli, brussel sprouts, cabbages

2 potatoes, carrots, beetroots

*3 spinach, sorrel, marinated vegetables*

4 sour pickles, sour marinated tomatoes

**2. How often do you consume sour pickles:**

1 never

2 less than once a week

3 1-2 times per week

*4 3-5 times per week*

*5 every day*

*6 a few times a day*

**3. Which salad sauce do you find the most tasty (mark one):**

1 soy sauce

2 lemon juice

*3 salt*

4 pepper

**4. Do you add extra salt to dishes while eating:**

1 never

*2 a little, if the dish is not salty enough*

*3 almost always, without even tasting*

**SOUR TASTE**

**1. Which of these vegetable groups do you find the most tasty (mark one):**

1 broccoli, brussel sprouts, cabbages

2 potatoes, carrots, beetroots

3 spinach, sorrel, marinated vegetables

*4 sour pickles, sour marinated tomatoes*

**2. How often do you consume sauerkraut:**

1 never

2 less than once a week

3 1-2 times per week

*4 3-5 times per week*

*5 every day*

*6 a few times a day*

**3. Which of this fruit do you prefer:**

1 bitter ones (grapefruit group)

2 sweet ones (pears, bananas, sweet apples)

*3 sour ones (oranges, currants, cherries, lemons)*

**4. Which salad sauce do you find the most tasty (mark one):**

1 soy sauce

*2 lemon juice*

3 salt

4 pepper

**5. Which group of products do you prefer:**

1 milk and sweet dairy products (sweet cheese, sweet cottage cheese, sweet yoghurt);

*2 sour dairy products (kefir, butter milk, natural yoghurt);*

3 fermented cheese

**UMAMI TASTE**

**1. How often do you consume tomatoes:**

1 never

2 less than once a week

3 1-2 times per week

*4 3-5 times per week*

*5 every day*

*6 a few times a day*

**2. Do you like mushrooms:**

*1 yes*

2 no

**3. Which salad sauce do you find the most tasty (mark one):**

*1 soy sauce*

2 lemon juice

3 salt

4 pepper

**4. Which kind of meat do you prefer:**

*1 poultry*

2 beef

3 pork

**5. Which way of cooked meat do you prefer:**

1 boiled / stewed

2 fried / roast

*3 smoked*

**6. How often do you consume instant soups and stock cubes:**

1 never

2 less than once a week

3 1-2 times per week

*4 3-5 times per week*

*5 every day*

*6 a few times a day*

**7. Which group of products do you prefer:**

1 milk and sweet dairy products (sweet cheese, sweet cottage cheese, sweet yoghurt);

*2 sour dairy products (kefir, butter milk, natural yoghurt);*

3 fermented cheese
