# Supplementary material for: Association study of taste preference: Analysis in the Lithuanian population
Source: Food Sci Nutr. 2021 Jun 27;9(8):4310–21. doi: 10.1002/fsn3.2401 (PMC8358374; doi:10.1002/fsn3.2401)
Supplement: Supplementary file 2 — Table [file FSN3-9-4310-s001.docx]

**Table S2. Table of all statistically significant results of the association analysis between sour taste preference and SNPs**

| **Chr**^a^ | **SNP** | **A1**^b^ | **A2**^c^ | **Test** | **Aff**^d^ | **Unaff**^e^ | **P** | **P**^f^ | **Fisher**  **P** | **Fisher**  **P**^g^ |
| --- | --- | --- | --- | --- | --- | --- | --- | --- | --- | --- |
| 10 | rs12360462 | G | A | Geno^h^ | 11/12/9 | 49/122/87 | 0.1277 |  | 0.1424 |  |
|  |  |  |  | Trend^i^ | 34/30 | 220/296 | 0.1215 |  | 0.1215 |  |
|  |  |  |  | Allelic^j^ | 34/30 | 220/296 | 0.1106 | 0.09489 | 0.1413 | 0.1022 |
|  |  |  |  | Dom^k^ | 23/9 | 171/87 | 0.5258 |  | 0.6908 |  |
|  |  |  |  | Rec^l^ | 11/21 | 49/209 | 0.04275 |  | 0.06144 |  |
| 12 | rs835592 | G | A | Geno | 8/16/8 | 31/137/90 | 0.1084 |  | 0.1183 |  |
|  |  |  |  | Trend | 32/32 | 199/317 | 0.06309 |  | 0.06309 |  |
|  |  |  |  | Allelic | 32/32 | 199/317 | 0.078 | 0.08949 | 0.0804 | 0.09149 |
|  |  |  |  | Dom | 24/8 | 168/90 | 0.2649 |  | 0.3243 |  |
|  |  |  |  | Rec | 8/24 | 31/227 | 0.04229 |  | 0.05392 |  |
| 12 | rs2272391 | A | G | Geno | 9/15/8 | 34/146/78 | 0.08062 |  | 0.103 |  |
|  |  |  |  | Trend | 33/31 | 214/302 | 0.09777 |  | 0.09777 |  |
|  |  |  |  | Allelic | 33/31 | 214/302 | 0.1236 | 0.06454 | 0.1408 | 0.05534 |
|  |  |  |  | Dom | 24/8 | 180/78 | 0.541 |  | 0.6824 |  |
|  |  |  |  | Rec | 9/23 | 34/224 | 0.02482 |  | 0.03424 |  |
| 12 | rs7305558 | A | G | Geno | 1/1/0 | 0/12/23 | – |  | 0.01952 |  |
|  |  |  |  | Trend | 3/1 | 12/58 | 0.003391 |  | 0.003391 |  |
|  |  |  |  | Allelic | 3/1 | 12/58 | 0.005118 | 0.005 | 0.02452 | 0.0142 |
|  |  |  |  | Dom | 2/0 | 12/23 | – |  | 0.1366 |  |
|  |  |  |  | Rec | 1/1 | 0/35 | – |  | 0.05405 |  |
| 16 | rs16973500 | A | G | Geno | 1/16/15 | 16/74/168 | – |  | 0.04991 |  |
|  |  |  |  | Trend | 18/46 | 106/410 | 0.1787 |  | 0.1787 |  |
|  |  |  |  | Allelic | 18/46 | 106/410 | 0.1629 | 0.1884 | 0.1948 | 0.08314 |
|  |  |  |  | Dom | 17/15 | 90/168 | – |  | 0.05249 |  |
|  |  |  |  | Rec | 1/31 | 16/242 | – |  | 0.7042 |  |
| 16 | rs9925415 | A | G | Geno | 2/22/8 | 69/116/73 | – |  | 0.009745 |  |
|  |  |  |  | Trend | 26/38 | 254/262 | 0.2044 |  | 0.2044 |  |
|  |  |  |  | Allelic | 26/38 | 254/262 | 0.1941 | 0.2291 | 0.2327 | 0.01645 |
|  |  |  |  | Dom | 24/8 | 185/73 | – |  | 0.8354 |  |
|  |  |  |  | Rec | 2/30 | 69/189 | – |  | 0.0085 |  |
| 16 | rs9928317 | C | A | Geno | 2/20/8 | 69/110/71 | – |  | 0.01551 |  |
|  |  |  |  | Trend | 24/36 | 248/252 | 0.1742 |  | 0.1742 |  |
|  |  |  |  | Allelic | 24/36 | 248/252 | 0.1598 | 0.1651 | 0.1734 | 0.02295 |
|  |  |  |  | Dom | 22/8 | 179/71 | – |  | 1 |  |
|  |  |  |  | Rec | 2/28 | 69/181 | – |  | 0.01304 |  |
| 16 | rs4788592 | A | G | Geno | 1/20/11 | 51/119/88 | – |  | 0.03139 |  |
|  |  |  |  | Trend | 22/42 | 221/295 | 0.1995 |  | 0.1995 |  |
|  |  |  |  | Allelic | 22/42 | 221/295 | 0.196 | 0.2151 | 0.2273 | 0.04405 |
|  |  |  |  | Dom | 11/21 | 170/88 | – |  | 1 |  |
|  |  |  |  | Rec | 3/29 | 51/207 | – |  | 0.02466 |  |
| 17 | rs16589 | A | G | Geno | 0/17/15 | 33/95/130 | – |  | 0.02911 |  |
|  |  |  |  | Trend | 17/47 | 161/355 | 0.4677 |  | 0.4677 |  |
|  |  |  |  | Allelic | 17/47 | 161/355 | 0.4479 | 0.4506 | 0.4767 | 0.05764 |
|  |  |  |  | Dom | 17/15 | 128/130 | – |  | 0.8516 |  |
|  |  |  |  | Rec | 0/32 | 33/225 | – |  | 0.03378 |  |
| 17 | rs16581 | A | G | Geno | 7/16/9 | 27/116/115 | 0.07725 |  | 0.08306 |  |
|  |  |  |  | Trend | 30/34 | 170/346 | 0.02645 |  | 0.02645 |  |
|  |  |  |  | Allelic | 30/34 | 170/346 | 0.02701 | 0.05604 | 0.0358 | 0.06559 |
|  |  |  |  | Dom | 23/9 | 143/115 | 0.07607 |  | 0.08926 |  |
|  |  |  |  | Rec | 7/25 | 27/231 | 0.05844 |  | 0.07644 |  |
| 17 | rs1988598 | A | G | Geno | 0/17/15 | 33/95/130 | – |  | 0.02911 |  |
|  |  |  |  | Trend | 17/47 | 161/355 | 0.4677 |  | 0.4677 |  |
|  |  |  |  | Allelic | 17/47 | 161/355 | 0.4479 | 0.4506 | 0.4767 | 0.05764 |
|  |  |  |  | Dom | 17/15 | 128/130 | – |  | 0.8516 |  |
|  |  |  |  | Rec | 0/32 | 33/225 | – |  | 0.03378 |  |
| 17 | rs4795742 | C | A | Geno | 7/16/9 | 27/120/111 | 0.09245 |  | 0.09252 |  |
|  |  |  |  | Trend | 30/34 | 174/342 | 0.03496 |  | 0.03496 |  |
|  |  |  |  | Allelic | 30/34 | 174/342 | 0.03765 | 0.07474 | 0.05121 | 0.07924 |
|  |  |  |  | Dom | 23/9 | 147/111 | 0.1065 |  | 0.1289 |  |
|  |  |  |  | Rec | 7/25 | 27/231 | 0.05844 |  | 0.07644 |  |
| 17 | rs4239237 | A | G | Geno | 4/10/18 | 46/124/88 | – |  | 0.05982 |  |
|  |  |  |  | Trend | 18/46 | 216/300 | 0.03831 |  | 0.03831 |  |
|  |  |  |  | Allelic | 18/46 | 216/300 | 0.03463 | 0.0382 | 0.0422 | 0.03185 |
|  |  |  |  | Dom | 14/18 | 170/88 | – |  | 0.01896 |  |
|  |  |  |  | Rec | 4/28 | 46/212 | – |  | 0.6209 |  |
| 17 | rs9916435 | A | C | Geno | 1/2/29 | 5/84/169 | – |  | 0.003468 |  |
|  |  |  |  | Trend | 4/60 | 94/422 | 0.01313 |  | 0.01313 |  |
|  |  |  |  | Allelic | 4/60 | 94/422 | 0.01596 | 0.0147 | 0.01299 | 0.005599 |
|  |  |  |  | Dom | 3/29 | 89/169 | – |  | 0.004048 |  |
|  |  |  |  | Rec | 1/31 | 5/253 | – |  | 0.5074 |  |
| 17 | rs16968024 | G | A | Geno | 10/9/13 | 20/112/126 | 0.000185 |  | 0.001195 |  |
|  |  |  |  | Trend | 29/35 | 152/364 | 0.01088 |  | 0.01088 |  |
|  |  |  |  | Allelic | 29/35 | 152/364 | 0.00982 | 0.00055 | 0.01442 | 0.001 |
|  |  |  |  | Dom | 19/13 | 132/126 | 0.3804 |  | 0.4545 |  |
|  |  |  |  | Rec | 10/22 | 20/238 | 3.84E-05 |  | 0.000428 |  |
| 17 | rs4239238 | A | G | Geno | 12/8/12 | 57/134/67 | 0.01463 |  | 0.01095 |  |
|  |  |  |  | Trend | 32/32 | 248/268 | 0.7719 |  | 0.7719 |  |
|  |  |  |  | Allelic | 32/32 | 248/268 | 0.7698 | 0.1147 | 0.7921 | 0.1208 |
|  |  |  |  | Dom | 20/12 | 191/67 | 0.167 |  | 0.2056 |  |
|  |  |  |  | Rec | 12/20 | 57/201 | 0.05354 |  | 0.07584 |  |
| 17 | rs9905111 | A | G | Geno | 14/12/6 | 56/133/69 | 0.02283 |  | 0.03364 |  |
|  |  |  |  | Trend | 40/24 | 245/271 | 0.02337 |  | 0.02337 |  |
|  |  |  |  | Allelic | 40/24 | 245/271 | 0.02339 | 0.01325 | 0.02467 | 0.0215 |
|  |  |  |  | Dom | 26/6 | 189/69 | 0.33 |  | 0.3971 |  |
|  |  |  |  | Rec | 14/18 | 56/202 | 0.005983 |  | 0.01413 |  |
| 17 | rs4541122 | G | A | Geno | 4/12/6 | 56/133/69 | 0.02283 |  | 0.03364 |  |
|  |  |  |  | Trend | 40/24 | 245/271 | 0.02337 |  | 0.02337 |  |
|  |  |  |  | Allelic | 40/24 | 245/271 | 0.02339 | 0.01325 | 0.02467 | 0.0215 |
|  |  |  |  | Dom | 26/6 | 189/69 | 0.33 |  | 0.3971 |  |
|  |  |  |  | Rec | 14/18 | 56/202 | 0.005983 |  | 0.01413 |  |
| 17 | rs4078690 | G | A | Geno | 0/1/31 | 4/64/190 | – |  | 0.01004 |  |
|  |  |  |  | Trend | 1/63 | 72/444 | 0.004435 |  | 0.004435 |  |
|  |  |  |  | Allelic | 1/63 | 72/444 | 0.00482 | 0.0025 | 0.002186 | 0.00105 |
|  |  |  |  | Dom | 1/31 | 68/190 | – |  | 0.001728 |  |
|  |  |  |  | Rec | 0/32 | 4/254 | – |  | 1 |  |
| 17 | rs7212444 | G | A | Geno | 13/13/6 | 44/130/84 | 0.005666 |  | 0.01077 |  |
|  |  |  |  | Trend | 39/25 | 218/298 | 0.004544 |  | 0.004544 |  |
|  |  |  |  | Allelic | 39/25 | 218/298 | 0.004526 | 0.0048 | 0.005061 | 0.007299 |
|  |  |  |  | Dom | 26/6 | 174/84 | 0.1113 |  | 0.1551 |  |
|  |  |  |  | Rec | 13/19 | 44/214 | 0.001552 |  | 0.003627 |  |
| 17 | rs9895456 | G | A | Geno | 13/12/7 | 49/135/74 | 0.01889 |  | 0.02972 |  |
|  |  |  |  | Trend | 38/26 | 233/283 | 0.02998 |  | 0.02998 |  |
|  |  |  |  | Allelic | 38/26 | 233/283 | 0.03151 | 0.0135 | 0.03403 | 0.01755 |
|  |  |  |  | Dom | 25/7 | 184/74 | 0.4182 |  | 0.5325 |  |
|  |  |  |  | Rec | 13/19 | 49/209 | 0.004872 |  | 0.0101 |  |
| 17 | rs1019410 | C | A | Geno | 8/10/14 | 17/114/127 | 0.001946 |  | 0.005728 |  |
|  |  |  |  | Trend | 26/38 | 148/368 | 0.0472 |  | 0.0472 |  |
|  |  |  |  | Allelic | 26/38 | 148/368 | 0.04924 | 0.0024 | 0.05967 | 0.0041 |
|  |  |  |  | Dom | 18/14 | 131/127 | 0.5589 |  | 0.5797 |  |
|  |  |  |  | Rec | 8/24 | 17/241 | 0.000465 |  | 0.002593 |  |
| 17 | rs1019412 | A | G | Geno | 3/4/25 | 22/88/148 | – |  | 0.03081 |  |
|  |  |  |  | Trend | 10/54 | 132/384 | 0.1021 |  | 0.1021 |  |
|  |  |  |  | Allelic | 10/54 | 132/384 | 0.0806 | 0.09824 | 0.09048 | 0.05569 |
|  |  |  |  | Dom | 7/25 | 110/148 | – |  | 0.03422 |  |
|  |  |  |  | Rec | 3/29 | 22/236 | – |  | 0.7461 |  |
| 17 | rs9889583 | A | G | Geno | 3/7/22 | 39/106/113 | – |  | 0.03546 |  |
|  |  |  |  | Trend | 13/51 | 184/332 | 0.02152 |  | 0.02152 |  |
|  |  |  |  | Allelic | 13/51 | 184/332 | 0.01448 | 0.02 | 0.0168 | 0.0177 |
|  |  |  |  | Dom | 10/22 | 145/113 | – |  | 0.008581 |  |
|  |  |  |  | Rec | 3/29 | 39/219 | – |  | 0.5934 |  |
| 17 | rs17249181 | G | A | Geno | 4/6/22 | 13/96/149 | – |  | 0.03752 |  |
|  |  |  |  | Trend | 14/50 | 122/394 | 0.7552 |  | 0.7552 |  |
|  |  |  |  | Allelic | 14/50 | 122/394 | 0.7528 | 0.7066 | 0.8759 | 0.159 |
|  |  |  |  | Dom | 10/22 | 109/149 | – |  | 0.2584 |  |
|  |  |  |  | Rec | 4/28 | 13/245 | – |  | 0.1034 |  |
| 17 | rs1007035 | C | A | Geno | 5/8/19 | 36/116/106 | 0.0855 |  | 0.08313 |  |
|  |  |  |  | Trend | 18/46 | 188/328 | 0.2046 |  | 0.2046 |  |
|  |  |  |  | Allelic | 18/46 | 188/328 | 0.1902 | 0.08834 | 0.2141 | 0.09519 |
|  |  |  |  | Dom | 13/19 | 152/106 | 0.04877 |  | 0.05871 |  |
|  |  |  |  | Rec | 5/27 | 36/222 | 0.798 |  | 0.7888 |  |
| 17 | rs1553499 | C | A | Geno | 7/7/18 | 18/111/128 | 0.004807 |  | 0.006899 |  |
|  |  |  |  | Trend | 21/43 | 147/367 | 0.486 |  | 0.486 |  |
|  |  |  |  | Allelic | 21/43 | 147/367 | 0.4839 | 0.01265 | 0.4695 | 0.0168 |
|  |  |  |  | Dom | 14/18 | 129/128 | 0.4917 |  | 0.5752 |  |
|  |  |  |  | Rec | 7/25 | 18/239 | 0.004772 |  | 0.01193 |  |
| 17 | rs2036535 | A | G | Geno | 11/15/6 | 50/126/82 | 0.09733 |  | 0.1072 |  |
|  |  |  |  | Trend | 37/27 | 226/290 | 0.03536 |  | 0.03536 |  |
|  |  |  |  | Allelic | 37/27 | 226/290 | 0.03366 | 0.08634 | 0.04519 | 0.08104 |
|  |  |  |  | Dom | 26/6 | 176/82 | 0.1304 |  | 0.156 |  |
|  |  |  |  | Rec | 11/21 | 50/208 | 0.04963 |  | 0.06444 |  |
| 17 | rs17836805 | C | A | Geno | 0/0/29 | 2/48/169 | – |  | 0.003942 |  |
|  |  |  |  | Trend | 0/58 | 52/386 | 0.004836 |  | 0.004836 |  |
|  |  |  |  | Allelic | 0/58 | 52/386 | 0.005546 | 0.005 | 0.002123 | 0.00195 |
|  |  |  |  | Dom | 0/29 | 50/169 | – |  | 0.002081 |  |
|  |  |  |  | Rec | 0/29 | 2/217 | – |  | 1 |  |
| 17 | rs2881564 | G | A | Geno | 8/12/11 | 26/123/109 | 0.03687 |  | 0.05733 |  |
|  |  |  |  | Trend | 28/34 | 175/341 | 0.07587 |  | 0.07587 |  |
|  |  |  |  | Allelic | 28/34 | 175/341 | 0.07963 | 0.02505 | 0.0912 | 0.02055 |
|  |  |  |  | Dom | 20/11 | 149/109 | 0.4702 |  | 0.5644 |  |
|  |  |  |  | Rec | 8/23 | 26/232 | 0.01022 |  | 0.01741 |  |
| 17 | rs11650439 | A | G | Geno | 2/5/25 | 1/50/207 | – |  | 0.04446 |  |
|  |  |  |  | Trend | 9/55 | 52/464 | 0.3251 |  | 0.3251 |  |
|  |  |  |  | Allelic | 9/55 | 52/464 | 0.327 | 0.3376 | 0.3847 | 0.0441 |
|  |  |  |  | Dom | 7/25 | 51/207 | – |  | 0.8151 |  |
|  |  |  |  | Rec | 2/30 | 1/257 | – |  | 0.03304 |  |
| 17 | rs9891235 | G | A | Geno | 6/17/9 | 20/113/125 | 0.03114 |  | 0.02869 |  |
|  |  |  |  | Trend | 29/35 | 153/363 | 0.009309 |  | 0.009309 |  |
|  |  |  |  | Allelic | 29/35 | 153/363 | 0.01087 | 0.0281 | 0.01473 | 0.01995 |
|  |  |  |  | Dom | 23/9 | 133/125 | 0.02962 |  | 0.03789 |  |
|  |  |  |  | Rec | 6/26 | 20/238 | 0.03997 |  | 0.05107 |  |
| 17 | rs7224103 | A | G | Geno | 0/4/28 | 7/86/163 | – |  | 0.02383 |  |
|  |  |  |  | Trend | 4/60 | 100/412 | 0.007351 |  | 0.007351 |  |
|  |  |  |  | Allelic | 4/60 | 100/412 | 0.009207 | 0.007549 | 0.00878 | 0.009799 |
|  |  |  |  | Dom | 4/28 | 93/163 | – |  | 0.008842 |  |
|  |  |  |  | Rec | 0/32 | 7/249 | – |  | 1 |  |
| 17 | rs4795800 | A | G | Geno | 7/8/17 | 13/106/139 | 0.001126 |  | 0.003738 |  |
|  |  |  |  | Trend | 22/42 | 132/384 | 0.1314 |  | 0.1314 |  |
|  |  |  |  | Allelic | 22/42 | 132/384 | 0.133 | 0.0024 | 0.1359 | 0.005 |
|  |  |  |  | Dom | 15/17 | 119/139 | 0.9359 |  | 1 |  |
|  |  |  |  | Rec | 7/25 | 13/245 | 0.000392 |  | 0.00285 |  |
| 17 | rs9914954 | A | G | Geno | 8/10/14 | 41/138/79 | 0.05822 |  | 0.04971 |  |
|  |  |  |  | Trend | 26/38 | 220/296 | 0.7534 |  | 0.7534 |  |
|  |  |  |  | Allelic | 26/38 | 220/296 | 0.7588 | 0.2569 | 0.79 | 0.2563 |
|  |  |  |  | Dom | 18/14 | 179/79 | 0.1334 |  | 0.1599 |  |
|  |  |  |  | Rec | 8/24 | 41/217 | 0.1946 |  | 0.2116 |  |
| 17 | rs8068295 | A | G | Geno | 1/7/24 | 12/111/135 | – |  | 0.04129 |  |
|  |  |  |  | Trend | 9/55 | 135/381 | 0.02672 |  | 0.02672 |  |
|  |  |  |  | Allelic | 9/55 | 135/381 | 0.03456 | 0.029 | 0.04491 | 0.02835 |
|  |  |  |  | Dom | 8/24 | 123/135 | – |  | 0.02261 |  |
|  |  |  |  | Rec | 1/31 | 12/246 | – |  | 1 |  |
| 17 | rs1865392 | A | G | Geno | 5/7/20 | 3/74/181 | – |  | 0.001134 |  |
|  |  |  |  | Trend | 17/47 | 80/436 | 0.02515 |  | 0.02515 |  |
|  |  |  |  | Allelic | 17/47 | 80/436 | 0.02535 | 0.0257 | 0.03256 | 0.0015 |
|  |  |  |  | Dom | 12/20 | 77/181 | – |  | 0.4177 |  |
|  |  |  |  | Rec | 5/27 | 3/255 | – |  | 0.000534 |  |
| 17 | rs1434586 | A | G | Geno | 8/7/17 | 24/104/130 | 0.01152 |  | 0.01568 |  |
|  |  |  |  | Trend | 23/41 | 152/364 | 0.3079 |  | 0.3079 |  |
|  |  |  |  | Allelic | 23/41 | 152/364 | 0.2867 | 0.0309 | 0.3127 | 0.0243 |
|  |  |  |  | Dom | 15/17 | 128/130 | 0.7702 |  | 0.8521 |  |
|  |  |  |  | Rec | 8/24 | 24/234 | 0.007513 |  | 0.01421 |  |
| 17 | rs11653827 | A | G | Geno | 1/9/22 | 1/24/233 | – |  | 0.001637 |  |
|  |  |  |  | Trend | 11/53 | 26/490 | 0.000247 |  | 0.000247 |  |
|  |  |  |  | Allelic | 11/53 | 26/490 | 0.000176 | 0.00065 | 0.001069 | 0.0014 |
|  |  |  |  | Dom | 10/22 | 25/233 | – |  | 0.001764 |  |
|  |  |  |  | Rec | 1/31 | 1/257 | – |  | 0.2089 |  |
| 17 | rs317397 | G | A | Geno | 6/7/19 | 20/109/128 | 0.02589 |  | 0.02276 |  |
|  |  |  |  | Trend | 19/45 | 149/365 | 0.9087 |  | 0.9087 |  |
|  |  |  |  | Allelic | 19/45 | 149/365 | 0.9075 | 0.09984 | 0.8849 | 0.07979 |
|  |  |  |  | Dom | 13/19 | 129/128 | 0.3072 |  | 0.3514 |  |
|  |  |  |  | Rec | 6/26 | 20/237 | 0.04088 |  | 0.05183 |  |
| 17 | rs317401 | A | G | Geno | 6/7/19 | 20/108/130 | 0.02793 |  | 0.02613 |  |
|  |  |  |  | Trend | 19/45 | 148/368 | 0.8691 |  | 0.8691 |  |
|  |  |  |  | Allelic | 19/45 | 148/368 | 0.8669 | 0.09874 | 0.884 | 0.08064 |
|  |  |  |  | Dom | 13/19 | 128/130 | 0.3373 |  | 0.3555 |  |
|  |  |  |  | Rec | 6/26 | 20/238 | 0.03997 |  | 0.05107 |  |
| 17 | rs4795831 | A | G | Geno | 4/4/24 | 7/68/183 | – |  | 0.01593 |  |
|  |  |  |  | Trend | 12/52 | 82/434 | 0.5743 |  | 0.5743 |  |
|  |  |  |  | Allelic | 12/52 | 82/434 | 0.5583 | 0.5507 | 0.5893 | 0.04395 |
|  |  |  |  | Dom | 8/24 | 75/183 | – |  | 0.8359 |  |
|  |  |  |  | Rec | 4/28 | 7/251 | – |  | 0.02326 |  |
| 17 | rs317413 | A | G | Geno | 6/7/19 | 20/109/128 | 0.02589 |  | 0.02276 |  |
|  |  |  |  | Trend | 19/45 | 149/365 | 0.9087 |  | 0.9087 |  |
|  |  |  |  | Allelic | 19/45 | 149/365 | 0.9075 | 0.09904 | 0.8849 | 0.08139 |
|  |  |  |  | Dom | 13/19 | 129/128 | 0.3072 |  | 0.3514 |  |
|  |  |  |  | Rec | 6/26 | 20/237 | 0.04088 |  | 0.05183 |  |
| 17 | rs317418 | G | A | Geno | 2/23/7 | 38/108/112 | – |  | 0.006445 |  |
|  |  |  |  | Trend | 27/37 | 184/332 | 0.3116 |  | 0.3116 |  |
|  |  |  |  | Allelic | 27/37 | 184/332 | 0.3058 | 0.3117 | 0.3357 | 0.0386 |
|  |  |  |  | Dom | 25/7 | 146/112 | – |  | 0.02195 |  |
|  |  |  |  | Rec | 2/30 | 38/220 | – |  | 0.2774 |  |
| 17 | rs17783779 | G | A | Geno | 1/19/12 | 16/90/152 | – |  | 0.0269 |  |
|  |  |  |  | Trend | 21/43 | 122/394 | 0.1064 |  | 0.1064 |  |
|  |  |  |  | Allelic | 21/43 | 122/394 | 0.1084 | 0.1088 | 0.1239 | 0.03035 |
|  |  |  |  | Dom | 20/12 | 106/152 | – |  | 0.02399 |  |
|  |  |  |  | Rec | 1/31 | 16/242 | – |  | 0.7042 |  |
| 17 | rs4451997 | A | G | Geno | 13/13/6 | 56/135/67 | 0.05972 |  | 0.07824 |  |
|  |  |  |  | Trend | 39/25 | 247/269 | 0.04621 |  | 0.04621 |  |
|  |  |  |  | Allelic | 39/25 | 247/269 | 0.04855 | 0.03725 | 0.06288 | 0.04185 |
|  |  |  |  | Dom | 26/6 | 191/67 | 0.3748 |  | 0.5173 |  |
|  |  |  |  | Rec | 13/19 | 56/202 | 0.01775 |  | 0.0263 |  |
| 17 | rs1003916 | A | G | Geno | 2/10/20 | 4/57/197 | – |  | 0.06904 |  |
|  |  |  |  | Trend | 14/50 | 65/451 | 0.0431 |  | 0.0431 |  |
|  |  |  |  | Allelic | 14/50 | 65/451 | 0.04124 | 0.0478 | 0.05214 | 0.06814 |
|  |  |  |  | Dom | 12/20 | 61/197 | – |  | 0.1282 |  |
|  |  |  |  | Rec | 2/30 | 4/254 | – |  | 0.1335 |  |
| 17 | rs11080254 | G | A | Geno | 9/12/11 | 37/140/81 | 0.08143 |  | 0.0914 |  |
|  |  |  |  | Trend | 30/34 | 214/302 | 0.3905 |  | 0.3905 |  |
|  |  |  |  | Allelic | 30/34 | 214/302 | 0.409 | 0.09569 | 0.4231 | 0.1039 |
|  |  |  |  | Dom | 21/11 | 177/81 | 0.7326 |  | 0.8406 |  |
|  |  |  |  | Rec | 9/23 | 37/221 | 0.0441 |  | 0.06787 |  |
| 17 | rs16589 | A | G | Geno | 0/17/15 | 33/95/130 | – |  | **0.02911** |  |
|  |  |  |  | Trend | 17/47 | 161/355 | 0.4677 |  | 0.4677 |  |
|  |  |  |  | Allelic | 17/47 | 161/355 | 0.4479 | 0.4506 | 0.4767 | 0.05764 |
|  |  |  |  | Dom | 17/15 | 128/130 | – |  | 0.8516 |  |
|  |  |  |  | Rec | 0/32 | 33/225 | – |  | **0.03378** |  |

a — chromosome; b — allele 1; c — allele 2; d — distribution of alleles or genotypes in the case group; e — distribution of alleles or genotypes in the control group; f — empirical *P*-value for χ^2^ test of independence (permutation test based on the most significant result of allelic dominant and recessive models); g — empirical *P*-value for Fisher’s Exact Test (permutation test based on the most significant result of allelic dominant and recessive models); h — basic model: genotypic; i — additive model: Cochran–Armitage trend; j — basic model: allelic; k — additive model: dominant; l — additive model: recessive; m — no data available.
